# Synchronous monitoring of brain‐heart electrophysiology using heart rate variability coupled with rapid quantitative electroencephalography in orthostatic hypotension patients with α‐synucleinopathies: Rapid prediction of orthostatic hypotension and preliminary exploration of brain stimulation therapy

**DOI:** 10.1111/cns.14571

**Published:** 2024-02-07

**Authors:** Lin Lin, Yingzhe Cheng, Peilin Huang, Jiejun Zhang, Jiahao Zheng, Xiaodong Pan

**Affiliations:** ^1^ Department of Neurology, Center for Cognitive Neurology Fujian Medical University Union Hospital Fuzhou City China; ^2^ Fujian Institute of Geriatrics Fujian Medical University Union Hospital Fuzhou City China; ^3^ Institute of Clinical Neurology Fujian Medical University Fuzhou City China; ^4^ Fujian Key Laboratory of Molecular Neurology Fujian Medical University Fuzhou City China; ^5^ Center for Geriatrics Hainan General Hospital Haikou City Hainan Province China

**Keywords:** heart rate variability, orthostatic hypotension, quantitative electroencephalography, α‐Synucleinopathy

## Abstract

**Background:**

In α‐synucleinopathies, the dysfunction of the autonomic nervous system which typically manifests as orthostatic hypotension (OH) often leads to severe consequences and poses therapeutic challenges. This study aims to discover the brain‐cardiac electrophysiological changes in OH patients with α‐synucleinopathies using the rapid quantitative electroencephalography (qEEG) coupled with heart rate variability (HRV) technique to identify rapid, noninvasive biomarkers for early warning and diagnosis, as well as shed new light on complementary treatment approaches such as brain stimulation targets.

**Methods:**

In this study, 26 subjects of α‐synucleinopathies with OH (α‐OH group), 21 subjects of α‐synucleinopathies without OH (α‐NOH group), and 34 healthy controls (control group) were included from September 2021 to August 2023 (NCT05527067). The heart rate‐blood pressure variations in supine and standing positions were monitored, and synchronization parameters of seated resting‐state HRV coupled with qEEG were collected. Time‐domain and frequency‐domain of HRV measures as well as peak frequency and power of the brainwaves were extracted. Differences between these three groups were compared, and correlations between brain‐heart parameters were analyzed.

**Results:**

The research results showed that the time‐domain parameters such as MxDMn, pNN50, RMSSD, and SDSD of seated resting‐state HRV exhibited a significant decrease only in the α‐OH group compared to the healthy control group (*p* < 0.05), while there was no significant difference between the α‐NOH group and the healthy control group. Several time‐domain and frequency‐domain parameters of seated resting‐state HRV were found to be correlated with the blood pressure changes within the first 5 min of transitioning from supine to standing position (*p* < 0.05). Differences were observed in the power of beta1 waves (F4 and Fp2) and beta2 waves (Fp2 and F4) in the seated resting‐state qEEG between the α‐OH and α‐NOH groups (*p* < 0.05). The peak frequency of theta waves in the Cz region also showed a difference (*p* < 0.05). The power of beta2 waves in the Fp2 and F4 brain regions correlated with frequency‐domain parameters of HRV (*p* < 0.05). Additionally, abnormal electrical activity in the alpha, theta, and beta1 waves was associated with changes in heart rate and blood pressure within the first 5 min of transitioning from supine to standing position (*p* < 0.05).

**Conclusion:**

Rapid resting‐state HRV with certain time‐domain parameters below normal levels may serve as a predictive indicator for the occurrence of orthostatic hypotension (OH) in patients with α‐synucleinopathies. Additionally, the deterioration of HRV parameters correlates with synchronous abnormal qEEG patterns, which can provide insights into the brain stimulation target areas for OH in α‐synucleinopathy patients.

## Graphical abstract

This study achieved the prediction of OH occurrence in patients with α‐synucleinopathies using the ultra‐short‐term resting heart rate variability (HRV). It also utilized HRV coupled with quantitative electroencephalography technology to explore the brain‐heart correlation, shedding light on identifying the target areas in brain stimulation therapy.

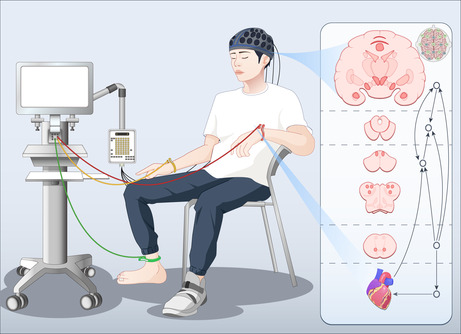

## 1 INTRODUCTION

α‐Synucleinopathies are a group of neurodegenerative diseases characterized by the misfolding and aggregation of α‐synuclein (α‐syn) protein, leading to the formation of intracellular inclusions. Common examples include Parkinson's disease (PD), dementia with Lewy bodies (DLB), and multiple system atrophy (MSA).^1^, ^2^ The non‐motor symptoms of these diseases have received increasing attention from researchers.^3^ Among them, autonomic dysfunction with orthostatic hypotension (OH) as the main manifestation can cause cerebral hypoperfusion and other adverse consequences,^4^, ^5^ even affecting survival rates.^6^ OH may occur before the onset of orthostatic symptoms and requires treatment.^7^ Therefore, it is of great significance to discover non‐invasive identification markers to facilitate early treatment. However, patients with severe motor dysfunction such as bradykinesia, difficulty maintaining balance, easy falls, limb weakness, and even paralysis,^8^, ^9^ as well as the severe OH‐induced symptoms such as orthostatic headaches, visual blackout, and even syncope,^10^ and the inability to independently follow instructions due to severe cognitive impairment,^11^, ^12^, ^13^ undergoing blood pressure and heart rate measurements through rapid positional changes (from supine to standing) for diagnosing OH can be extremely distressing or challenging for patients with α‐synucleinopathies. Therefore, this study aims to find a comfortable and short‐duration examination method in a seated position that can predict the occurrence of OH and explore more treatment options for OH based on this method.

It was believed that impaired resting‐state heart rate variability (HRV) closely correlated with the occurrence of OH, which may indicate a compensation failure in the orthostatic heart beat regulation^14^ and serve as a warning for worse prognosis in α‐synucleinopathies.^15^, ^16^ HRV is an indicator that measures the fluctuation of cardiac interbeat intervals over time, which is mediated by the interaction between the two branches of the autonomic nervous system: the sympathetic nervous system (SNS) and the parasympathetic nervous system (PNS) that controls the sinus node and ventricular walls.^17^ It is widely regarded as a representative measure of autonomic nervous system function.^18^ It has been proven that patients with PD, MSA, and DLB exhibit a decrease in time‐domain and frequency‐domain parameters of resting‐state HRV.^19^, ^20^, ^21^ However, the predictive ability of rapid resting‐state HRV for changes in OH relative to normal levels in α‐synucleinopathies is not yet clear. With the advancement of technology, the efficient acquisition and analysis of ultra‐short‐term resting‐state HRV in a comfortable position for patients have gradually shortened the duration.^22^, ^23^, ^24^ This greatly facilitates the measurement of blood pressure in supine‐to‐standing positions for patients with mobility difficulties or poor positional cooperation. However, due to the wide variety of HRV parameters,^25^ there is an urgent need to select meaningful parameters for OH patients with α‐synucleinopathies, which is also one of the objectives of this study.

Currently, the treatment options for α‐synucleinopathies patients with OH are limited, mainly maintaining blood pressure through medications. Early treatment can delay disease progression and improve the quality of life.^26^ Some new brain stimulation techniques have been developed for regulating motor and cognitive functions, but the treatment of OH is often overlooked.^27^, ^28^, ^29^, ^30^, ^31^ Electroencephalography (EEG) is widely used for exploring brain stimulation treatment sites and monitoring therapeutic efficacy.^32^, ^33^ Rapid quantitative electroencephalography (qEEG), as a noninvasive, radiation‐free, and convenient technique, can quantitatively analyze the dynamic electrical signal characteristics of the brain, serving as an auxiliary examination in the early warning, diagnosis, and differentiation of neurodegenerative diseases.^34^ Previous qEEG studies revealed an increase in slow activities in PD patients.^35^ It has been also found that delta or theta power correlating with cognitive impairments in PD‐related cognitive impairments,^36^ while beta power changes correlate with the severity of motor impairments in PD patients.^37^ Therefore, we also hope to discover new brain target areas or circuits to advance the treatment of OH in these types of diseases and to reflect the effects through real‐time resting‐state monitoring of HRV. This idea stems from the longstanding interest in the connection between neural activity in the heart and brain since the 19th century, which has been confirmed in anatomy and physiology studies.^38^ Since the 20th century, brain electrical activity has been found to have concurrent and feedback connections with peripheral autonomic activity, particularly cardiac activity.^39^, ^40^ This has also been observed in patients with PD.^41^ Postmortem studies of PD have shown the accumulation of Lewy bodies and neuronal loss in brain structures, including regions involved in HRV control, such as the frontal lobe,^42^ insula, anterior cingulate cortex, amygdala, hypothalamus, and brainstem nuclei.^43^ Meanwhile, HRV also serves as an alternative parameter to assess the complex interactions between brain tissue and the cardiovascular system.^17^, ^44^ Building upon the aforementioned research, in this study, qEEG was coupled with HRV in real time to monitor the brain‐heart interplay in OH patients with α‐synucleinopathies to identify the brain regions that are potentially involved in regulating autonomic function, particularly cardiovascular function, under pathological conditions.

We hypothesize that, under the condition of having equipment capable of real‐time monitoring of synchronous brain‐heart electrical activity, lower rapid resting‐state HRV parameters compared to normal levels can reflect the occurrence of OH in patients with α‐synucleinopathies. Additionally, the deterioration of their HRV parameters is believed to be correlated with synchronous abnormal EEG activity, which may indicate the brain stimulation target areas for OH in α‐synucleinopathies.

## 2 METHODS

### 2.1 Participants

**FIGURE 1:**
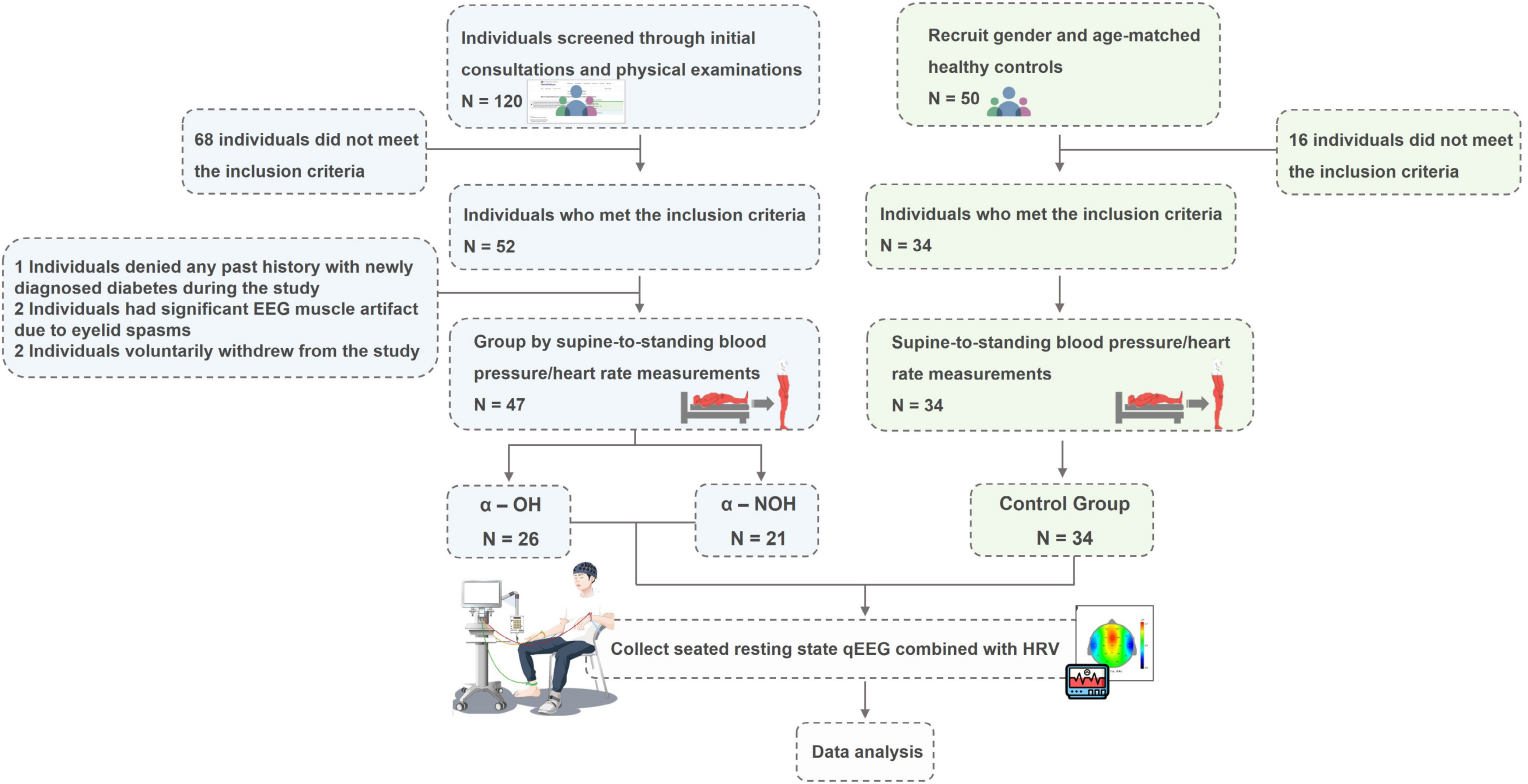
Standardized enrollment process diagram.

The standard enrollment process is illustrated in Figure 1. From September 2021 to June 2023, patients who came for the first visit at the Department of Neurology, Fujian Medical University Union Hospital, China, were recruited. A preliminary screening was conducted, resulting in 120 clinically suspected patients with α‐synucleinopathies. The inclusion criteria consisted of: age between 35 and 80 years old; PD, MSA, and DLB diagnosed based on the 2015 Movement Disorder Society clinical diagnostic criteria for PD,^45^ second consensus statement on the diagnosis of MSA (2008),^46^ and diagnosis and management of DLB (2017)^47^; willingness to cooperate with the study and sign informed consent forms. Exclusion criteria included confirmed confounding factors affecting HRV, such as cardio‐cerebral‐vascular diseases, diabetes, and malignant tumors; current use of medications affecting heart rhythms and brain electricity; current use of all antihypertensives and vasopressors affecting OH assessment; and poor compliance. The inclusion and exclusion of participants were performed by one neurology professor with 20 years of clinical experience. After exclusions, 52 patients met the inclusion criteria and were included in the study. Among these participants, 2 patients voluntarily withdrew from this study, 1 patient was excluded due to newly diagnosed diabetes during the course of the study, and 2 patients with eyelid muscle tension disorder were unable to cooperate during resting‐state EEG tests. Eventually, 47 patients with α‐synucleinopathies were enrolled (see Table S1 for more case data). All patients came for the first visit and underwent assessment, grouping, and collection of all study parameters immediately before standard clinical treatment. All patients did not receive sedative‐hypnotic drugs, anxiolytics, antidepressants, or antipsychotics. All participants provided demographic information, medical history, and a list of medications. Based on strict BP measurement and using the classic OH diagnostic criteria, these patients were divided into the α‐OH group (*N* = 26) and the α‐NOH group (*N* = 21).^48^

Additionally, a control group comprising 34 healthy individuals was included, and their gender and age were matched with the patients. The inclusion criteria for the healthy control group are as follows: male or female; aged 35–80 years old; willingness to cooperate with the study and sign informed consent forms. The exclusion criteria include the use of any medication or alcohol within the past 3 months, as well as any other form of medical intervention.

### 2.2 Ethics and informed consent

This program was approved by the Ethics Committee of FJMUUH (2020YF004‐01). This study was conducted based on the Cohort‐PDS cohort (NCT05527067, https://clinicaltrials.gov). The whole process was completed in accordance with the Declaration of Helsinki (1975) and the National Statement on Ethical Conduct in Research Involving Humans (1999). All authors had no conflicts of interest with this study.

### 2.3 Acquisition of orthostatic HR‐BP and symptoms data

All patients were placed in a fixed, independent, and quiet enclosed evaluation room and remained supine for 20 min before their BP and HR measurements were taken. Automated equipment (Omron‐HEM‐907 XL; Omron Healthcare) was used to collect upper arm blood pressure and heart rate (pulse rate) measurements. Simultaneously, the mean arterial pressure (MAP) was calculated. Patients were instructed to stand up quickly and without support, and their BP and HR were measured at 0, 1, 3, and 5 min, respectively. The differences in the patients' HR, MAP, systolic blood pressure (SBP), and diastolic blood pressure (DBP) at 0, 1, 3, and 5 min compared to the supine position were calculated. These differences were represented by ΔHR, ΔMAP, ΔSBP, and ΔDBP, respectively (negative values indicate a decrease in HR or BP compared to the supine position).

### 2.4 Acquisition of rapid resting‐state brain‐heart electrophysiological data

The examinations were performed in a fixed, independent, and quiet enclosed room. Prior to the tests, all subjects were required to have had a normal night's sleep of 6–8 h, without sleep deprivation or insomnia. The data collection was performed at the same fixed time in the morning on an empty stomach. Participants were instructed to sit quietly and rest for 5 min before the tests. They were asked to close their eyes and remain awake, while wearing noise‐canceling headphones. Under the aforementioned conditions, a 3‐min resting‐state EEG and ECG were recorded synchronously.

#### 2.4.1 Cardiac electricity acquisition

Limbs leads (aVR, aVL, and aVF) were connected to the patient's left and right wrists and a single ankle to record the cardiac electricity. A 3‐min seated resting‐state ECG was simultaneously recorded with the EEG. The alternating current was set at 220 V, 50 Hz. The sampling rate was 2000 Hz. Butterworth Filter was used for filtering with the following settings: high‐pass filter at 0.15 Hz, low‐pass filter at 70 Hz, and notch filter at 50 Hz. The KARDi2/4‐B autonomic function mapping ECG system (NeuroMed, China) was employed to record and analyze the millivolt‐level signals of ECG oscillations. The system automatically calculates the average of the preceding three and subsequent three normal RR intervals and adds markers to clean the data and eliminate ectopic heartbeats. Using the built‐in Pan‐Tompkins algorithm, the system completes the detection of the time domain indices of HRV including MxDMn (the variation scope reflecting the degree of RR interval variability), pNN50 (percentage of adjacent intervals that varied by greater than 50 ms), RMSSD (the root mean square of successive differences), SDNN (the standard deviation of all NN intervals), SDSD (the standard deviation of the differences between consecutive NN intervals), and frequency domain indices (including TP (total power), HF (high frequency, power spectrum between 0.15 and 0.40 Hz), LF (low frequency, power spectrum between 0.04 and 0.15 Hz), VLF (very low frequency, power spectrum below 0.04 Hz), LF% (LF as a percentage of TP)).^49^

#### 2.4.2 Brain electricity acquisition

A 19‐electrode cap was placed on subject's head (the NVX52 scalp EEG acquisition device (NeuroMed, China)), with electrodes positioned at Fp1, Fp2, F3, F4, Fz, F7, F8, C3, C4, Cz, P3, P4, Pz, T3, T4, T5, T6, O1, and O2. A reference electrode was placed on the ear lobes (A1 and A2). A hardware amplifier (Medical Computer Systems Ltd., Russia) was used, employing a 52‐channel DC amplifier powered by direct current. The internal parameters include a 24‐bit A/D conversion, a sampling rate of 50 KHz, a common mode rejection ratio (CMRR) of not less than 110 dB, an input impedance of not less than 100 MOhm, and a noise level of not exceeding 1 μV. The alternating current was set at 220 V, 50 Hz. The sampling rate was set at 2000 Hz, with a high‐pass filter set at 0.5 Hz, a low‐pass filter at 30 Hz, and a notch filter at 50 Hz. Using the fast Fourier transform algorithm with a step size of 0.25 Hz, a Hann window, and a window length of 4 s, the output provides the frequency‐power spectrum of brain oscillations. EEG signals were collected, and brainwave oscillation frequency‐power was output, including delta (δ, 1.5–4 Hz), theta (θ, 4–7.5 Hz), alpha (α, 7.5–14 Hz), and beta1 (β1, 14–20 Hz), and beta2 (β2, 20–30 Hz). Considering that α waves reflect the most fundamental natural state of human brain tissue and are most prominent during awake, closed‐eye conditions, we designed a built‐in technology to calculate the dispersion of alpha peak frequencies in the EEG signals. The As value was defined as the asymmetric distribution value of CDα1 (the quotient of the normalized distribution value of α rhythm power and the total power of 7–13 Hz brainwaves).

### 2.5 Statistical analysis

The Shapiro‐Wilks W test was performed to determine whether the data followed a normal distribution. When the data did not follow a normal distribution, nonparametric tests were used. Continuous variables were described using median with interquartile range (IQR) for nonparametric data. Kruskal‐Wallis followed by Bonferroni post hoc analysis was used for multiple testing. The chi‐square test was used to analyze categorical variables. The Spearman's correlation was used for analyzing the correlation when the data are not normally distributed. Statistical analysis was performed using SPSS software (version 26.0, Chicago, Illinois, USA). Graphs were created using Prism software (version 9.0, GraphPad Prism, USA, https://www.graphpad.com/scientific‐software/prism/) and R software (version 4.2.3, R Core Team, New Zealand, http://www.r‐project.org/). The schematic diagram illustrating intergroup differences in EEG was drawn based on the Cartesian coordinates model.

## 3 RESULTS

### 3.1 Analysis of demographic distribution and postural BP changes

The analysis of sociodemographic and BP data in supine and standing positions in the α‐OH, α‐NOH, and control group is shown in Table 1. There were no significant differences in gender, age, and level of education among the groups. During the BP monitoring in supine and standing positions, the α‐OH group showed a significant decrease in MAP, SBP, and DBP compared to the α‐NOH and control groups within 5 min (*p* < 0.05).

**TABLE 1:** Demographic and BP data of subjects in standing and lying positions in the α‐OH, α‐NOH, and control group.

	α‐OH *N* = 26	α‐NOH *N* = 21	Control group *N* = 34	Comparison	*p*‐value
Gender (male, *n*)	17	7	21	Chi‐square	0.056; OH = NOH = Ctrl
Age, years*	70.5 (62.8 to 75.0)	67.0 (59.0 to 74.5)	68.0 (61.3 to 71.3)	Nonparametric tests	0.468; OH = NOH = Ctrl
Education, years	9.0 (6.0 to 14.0)	7.0 (5.0 to 11.0)	9.5 (8.0 to 12.0)	Nonparametric tests	0.125; OH = NOH = Ctrl
ΔMAP – 0 min, mmHg	−1.2 (−4.1 to 4.4)	14.3 (8.3 to 21.0)	15.7 (10.8 to 20.3)	Nonparametric tests	< 0.001; OH < NOH = Ctrl
ΔMAP – 1 min, mmHg	14.3 (9.6 to 19.7)	18.0 (12.7 to 22.0)	14.3 (10.9 to 18.5)	Nonparametric tests	0.262; OH = NOH = Ctrl
ΔMAP – 3 min, mmHg	9.5 (−7.5 to 13.8)	13.3 (9.8 to 19.5)	14.7 (11.7 to 18.1)	Nonparametric tests	< 0.001; OH < NOH = Ctrl
ΔMAP – 5 min, mmHg	−7.3 (−13.0 to −3.6)	0.7 (−3.2 to 5.0)	0.2 (−4.2 to 4.3)	Nonparametric tests	< 0.001; OH < NOH = Ctrl
ΔSBP – 0 min, mmHg	−22 (−29.0 to −20.8)	−1.0 (−7.0 to 5.0)	−0.5 (−4.3 to 5.0)	Nonparametric tests	< 0.001; OH < NOH = Ctrl
ΔSBP – 1 min, mmHg	−18 (−23.0 to −11.8)	2.0 (−3.0 to 6.5)	−1.0 (−6.0 to 5.3)	Nonparametric tests	< 0.001; OH < NOH = Ctrl
ΔSBP – 3 min, mmHg	−15.0 (−19.3 to −9.8)	−1.0 (−7.0 to 6.0)	−1.0 (−5.0 to 5.0)	Nonparametric tests	< 0.001; OH < NOH = Ctrl
ΔSBP – 5 min, mmHg	−13.5 (−20.0 to −7.8)	−1.0 (−6.5 to 9.5)	−2.0 (−7.3 to 2.5)	Nonparametric tests	< 0.001; OH < NOH = Ctrl
ΔDBP – 0 min, mmHg	−8.5 (−12.0 to −3.0)	1.0 (−2.5 to 6.5)	2.5 (−2.0 to 6.3)	Nonparametric tests	< 0.001; OH < NOH = Ctrl
ΔDBP – 1 min, mmHg	4.0 (−2.0 to 7.0)	1.0 (−1.0 to 3.0)	0.5 (−2.3 to 3.5)	Nonparametric tests	0.112; OH = NOH = Ctrl
ΔDBP – 3 min, mmHg	−3.5 (−9.0 to −9.8)	0 (−4.0 to 4.0)	1.5 (−3.0 to 6.5)	Nonparametric tests	< 0.001; OH < NOH = Ctrl
ΔDBP – 5 min, mmHg	−2.0 (−8.5 to 0)	−1.0 (−3.0 to 5.5)	2.0 (−2.3 to 6.8)	Nonparametric tests	0.002; OH < NOH = Ctrl

Abbreviation: Ctrl, control group.

*Note*: Continuous variables were described using median (IQR) for non‐parametric data in the tables.

### 3.2 Analysis of supine‐to‐standing HR changes and seated resting‐state HRV Indices

#### 3.2.1 Analysis of supine‐to‐standing HR changes

The results of HR changes during supine‐to‐standing BP monitoring are presented in Table 2. There were no significant differences in HR between the supine and standing positions within 5 min among the groups (*p* > 0.05). Only using the heart rate difference associated with changes in body position is not sufficient to differentiate between the three groups.

**TABLE 2:** Analysis results of HR changes of subjects in standing and lying positions.

	α‐OH *N* = 26	α‐NOH *N* = 21	Control group *N* = 34	Comparison	*p*‐value
ΔHR – 0 min, mmHg*	7.0 (2.0 to 12.5)	6.0 (1.5 to 12.0)	10.0 (4.8 to 14.3)	Nonparametric tests	0.281; OH = NOH = Ctrl
ΔHR – 1 min, mmHg	9.5 (2.3 to 12.0)	4.0 (1.0 to 8.0)	8.5 (1.8 to 12.0)	Nonparametric tests	0.106; OH = NOH = Ctrl
ΔHR – 3 min, mmHg	4.0 (1.0 to 8.3)	4.0 (−0.5 to 9.0)	6.0 (1.8 to 12.0)	Nonparametric tests	0.457; OH = NOH = Ctrl
ΔHR – 5 min, mmHg	5.0 (−9.0 to −1.0)	1.0 (−1.0 to 9.5)	5.5 (3.0 to 12.0)	Nonparametric tests	0.195; OH = NOH = Ctrl

Abbreviation: Ctrl, control group.

*Note*: Continuous variables were described using median (IQR) for non‐parametric data in the tables.

#### 3.2.2 Analysis of HRV monitoring results

**FIGURE 2:**
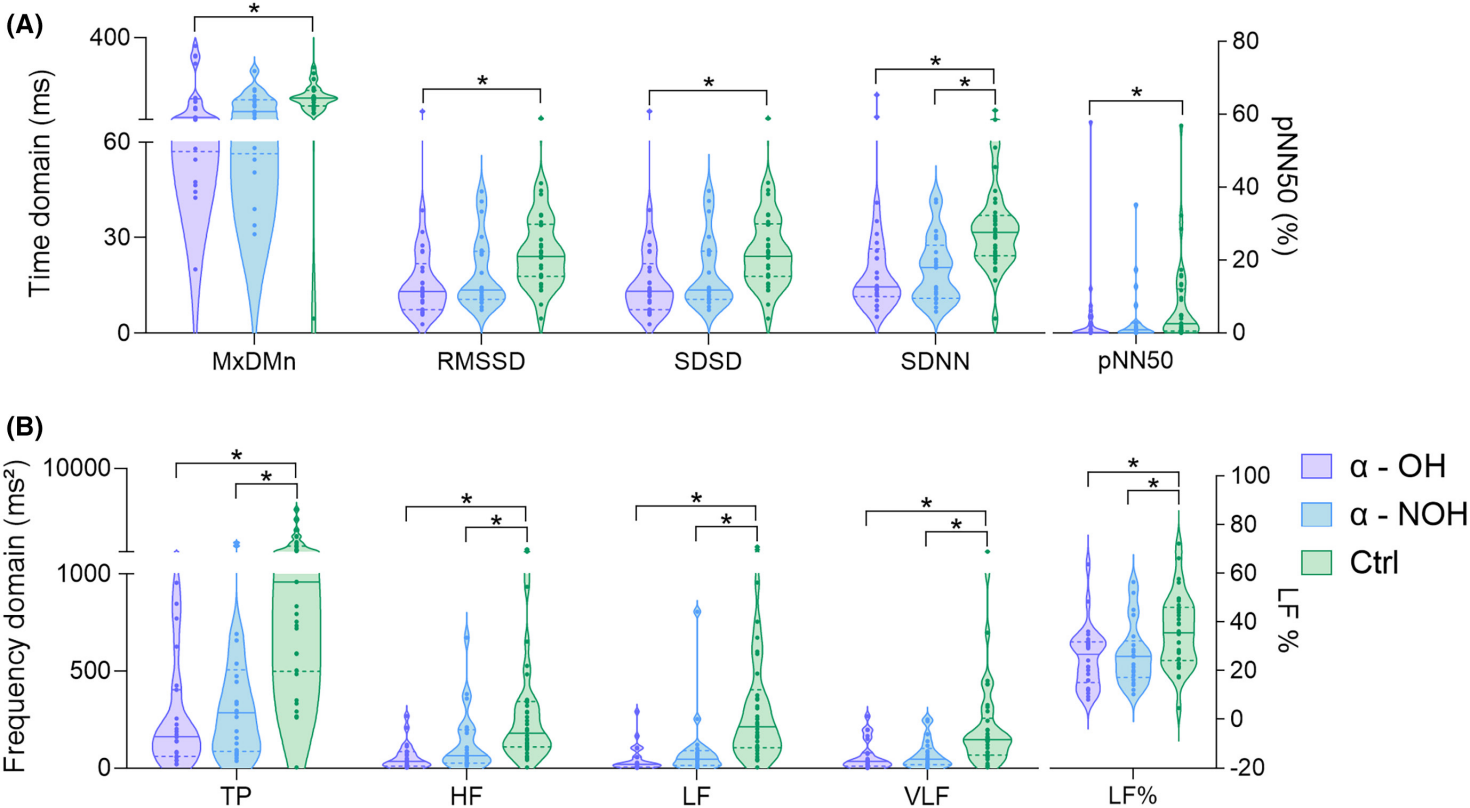
Comparison of HRV in the time domain (i, nonparametric tests) and the frequency domain (ii, nonparametric tests) between the α‐OH, α‐NOH, and control group.

To further explore changes in participants' cardiac electrical activities, HRV monitoring results from the three groups were analyzed (See Figure 2). In the time domain parameters (MxDMn, RMSSD, SDSD, and pNN50), the α‐OH group showed a significant decrease compared to the control group (*p* < 0.05). However, there were differences in both the time domain parameter of SDNN and the frequency domain parameters of TP, HF, LF, VLF, and LF in both the α‐NOH and α‐OH groups when compared to the normal levels (*p* < 0.05), so they are insufficient for identifying the occurrence of OH.

#### 3.2.3 Correlation analysis between seated resting‐state HRV and supine‐standing blood pressure differences

**FIGURE 3:**
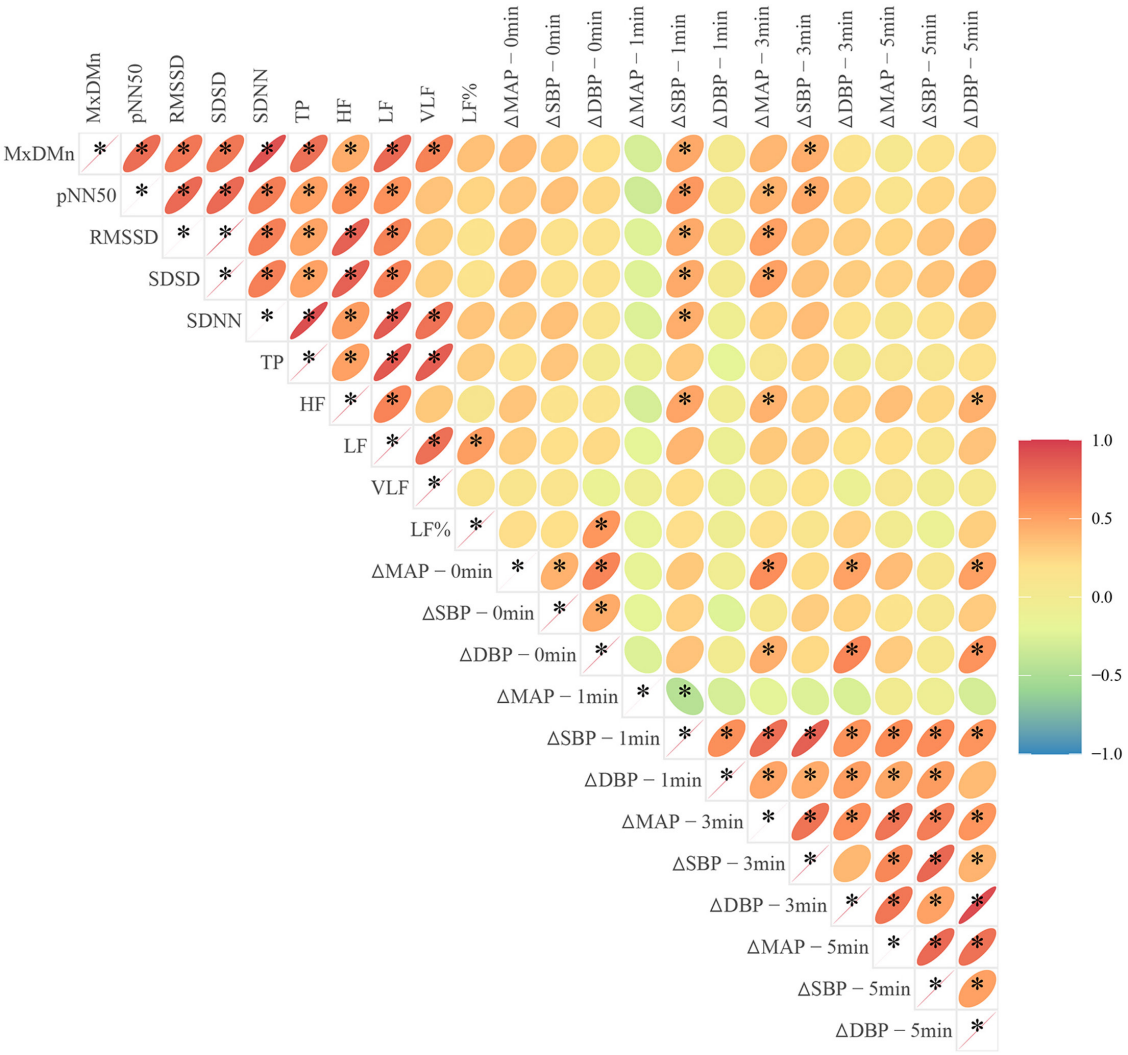
Spearman correlation analysis heatmap between resting‐state HRV and supine‐to‐standing blood pressure changes (the oval direction in the diagram indicates the positive or negative correlation; The rounder the shape and the lighter the color, the weaker the correlation).

The Spearman correlation analysis was performed between the parameters of seated resting‐state HRV in the α‐OH group and the supine‐standing blood pressure differences (as shown in Figure 3). The analysis results revealed significant correlations between the time domain parameters including MxDMn, pNN50, RMSSD, SDSD, SDNN, and the changes in supine‐standing blood pressure (primarily the differences between standing blood pressure and supine blood pressure after standing 1 and 3 min) (*p* < 0.05). It demonstrated that the more severe the orthostatic blood pressure drop within 3 min of standing (mainly reflected in ΔSBP and ΔMAP), the lower the mentioned time domain indices. Among the seated resting‐state frequency domain parameters, HF showed correlation with the overall changes in supine‐to‐standing blood pressure within 5 min, while LF% showed correlation with the supine‐to‐immediate standing blood pressure differences. Furthermore, there were significant correlations between the frequency domain and time domain parameters of HRV themselves (*p* < 0.05).

### 3.3 Between‐group qEEG comparative analysis

**FIGURE 4:**
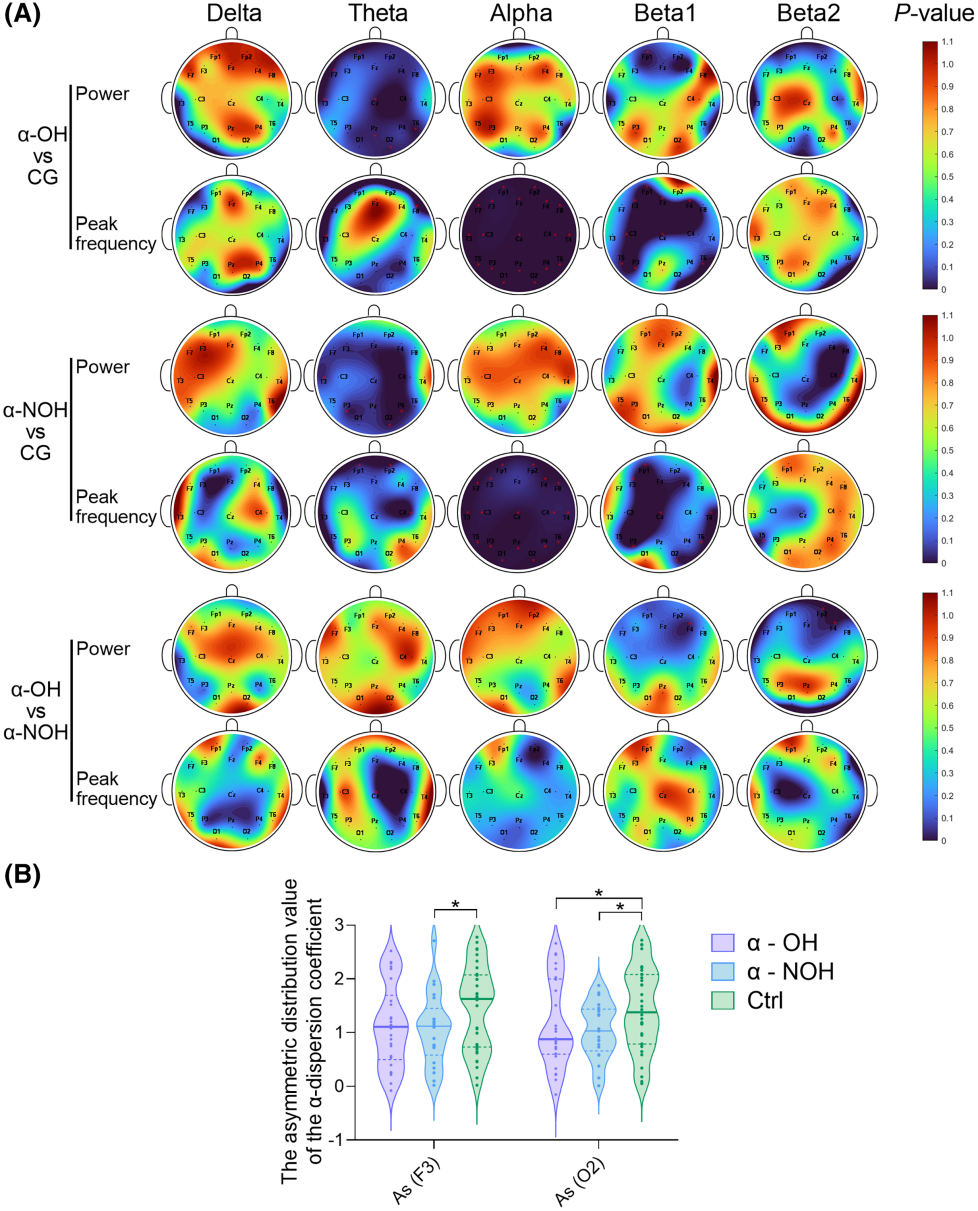
Comparison of qEEG data between the α‐OH, α‐NOH, and control group. (A) Differences in the power and PF of different brainwaves in different brain regions between two groups (nonparametric tests); (B) Comparison of the As value between these three groups (nonparametric tests).

Peak frequency and power of different waveforms in various brain regions were extracted by performing rapid qEEG, and, subsequently, a comparative analysis was conducted to examine the differences in EEG patterns between the groups, as shown in Figure 4A. Except for the slow wave—delta wave, which did not show significant differences between these groups. Except for that, various waveforms (theta, alpha, beta1, and beta2) in α‐synucleinopathies (with or without OH) exhibited significant differences in peak frequency or power (*p* < 0.05).

#### 3.3.1 Differences in the distribution and activity of alpha waves

As shown in Figure 4A, compared to the healthy control group, peak frequency of alpha waves, which are most prominent when a person is awake with their eyes closed, was significantly suppressed in the entire brain of patients in the α‐OH and α‐NOH groups (*p* < 0.05). However, there was no significant difference between the α‐OH and α‐NOH groups. As shown in Figure 4B, the calculation results indicated a significant decrease in the As value (representing the dispersion of the peak frequency of alpha waves) of the F3 brain region in the α‐NOH group compared to the control group (*p* < 0.05), but in the α‐OH group, compensatory and alleviating effects were observed. The As in the O2 brain region was significantly reduced in both the α‐OH and α‐NOH groups compared to the healthy control group (*p* < 0.05). However, some patients in the α‐OH group showed compensatory phenomena towards normal levels compared to the α‐NOH group.

#### 3.3.2 Distribution analysis of diversified brainwave frequency

The results demonstrated significant differences between the α‐OH and α‐NOH groups in power of beta1 waves (F4 and Fp2) and beta2 waves (Fp2 and F4) (*p* < 0.05) (Figure 4A). There were differences in peak frequency of theta waves (Cz) between the two groups (*p* < 0.05) (Figure 4A). It is worth noting that theta waves already changed in the α‐NOH group, which had not yet developed blood pressure abnormalities (*p* < 0.05). This change was also observed in the α‐OH group, compared to the control group and was widely distributed in anterior‐central‐posterior brain tissues (See Figure 4A).

### 3.4 Correlation analysis of qEEG and cardiovascular parameters and key brain region localization

**FIGURE 5:**
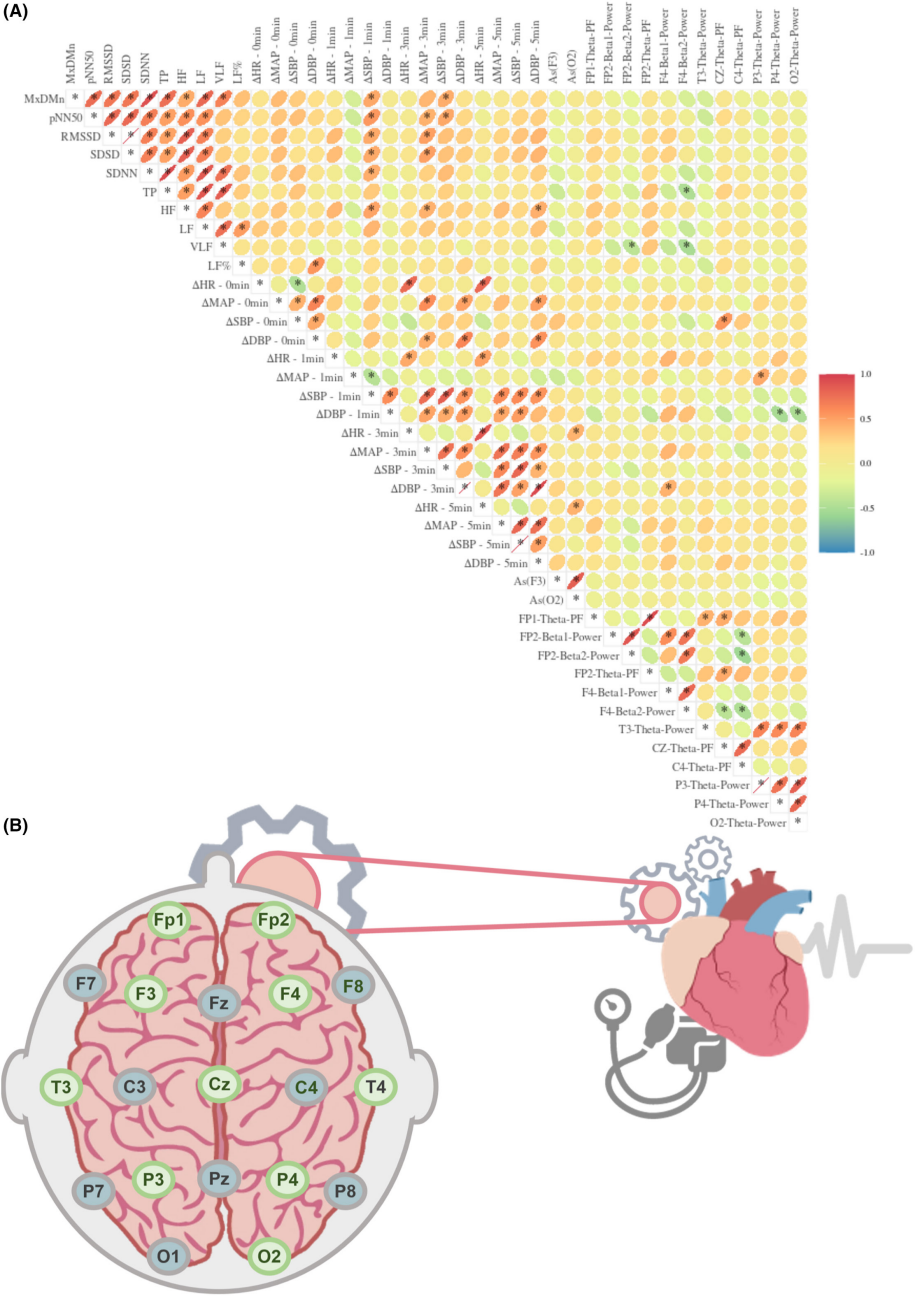
(A) The heatmap related to the coefficient of correlation of seated resting‐state qEEG parameters and resting‐state HRV parameters, supine‐to‐standing blood pressure and heart rate changes (the oval direction in the diagram indicates the positive or negative correlation; The rounder the shape and the lighter the color, the weaker the correlation); (B) Based on research findings, brain region schematic diagram of cardiovascular indices in OH patients with α‐synucleinopathies (green in the brain electrode region represents the brain regions where electrical activities are related to heart/BP and other brain regions with correlations). PF, peak frequency.

As shown in Figure 5A, a correlation analysis was conducted between qEEG and cardiovascular parameters, revealing synchronous electrical activity changes between the brain and heart. Key brain regions were identified that demonstrated synchronous electrical activity changes of them. Additionally, brain regions with meaningful results were analyzed and marked in Figure 5B (highlighted in green).

#### 3.4.1 Correlation between synchronously monitored seated resting‐state qEEG and HRV

Spearman correlation analysis was performed to examine the correlation between synchronously monitored seated resting‐state qEEG (parameters with positive results in the aforementioned Result 3) and HRV in the α‐OH group. The correlation coefficient heatmap is shown in Figure 5A. The positive results were primarily observed in the non‐dominant hemisphere, where the power of beta2 in the Fp2 brain region demonstrated a negative correlation with VLF in HRV (*p* < 0.05). Additionally, the power of beta2 waves in the F4 brain region exhibited a negative correlation with TP and VLF in HRV (*p* < 0.05).

#### 3.4.2 Correlation between seated resting‐state qEEG and supine‐to‐standing HR‐BP changes

Spearman correlation analysis was performed to examine the correlation between seated resting‐state qEEG (parameters with positive results in the aforementioned Result 3) supine‐to‐standing HR‐BP changes in the α‐OH group. The correlation coefficient heatmap is shown in Figure 5A. Abnormal electrical activity in theta and beta1 waves (distributed in F4, Cz, P3, P4 and O2) and the As value (representing the dispersion of the peak frequency of alpha waves) in the O2 region were correlated with ΔHR‐3 min, ΔHR‐5 min, ΔMAP‐1 min, ΔSBP‐0 min, ΔDBP‐1 min, and ΔDBP‐3 min (*p* < 0.05).

#### 3.4.3 Exploration of the correlations among electrical activity parameters of key brain regions

Correlation analysis was conducted among the electrical activity parameters, including peak frequency and power, of the aforementioned key brain regions. The results revealed correlations in electrical activity between multiple brain regions, with real‐time synchronous changes (*p* < 0.05). These correlations were predominantly observed in bilateral frontal lobes, bilateral parietal lobes, temporal lobes in the dominant hemisphere, and occipital lobes in the non‐dominant hemisphere.

## 4 DISCUSSION

In this study, a synchronized monitoring system combining non‐invasive and convenient ultra‐short‐term seated resting state HRV with qEEG was employed to rapidly collect and calculate brain‐heart electrical activities in study participants. This study is conducted based on the registered cohort of patients with α‐synucleinopathies to explore early biomarkers of autonomic dysfunction manifested as OH. We also assist in exploring the possible feedback‐negative feedback phenomenon, regulatory mechanisms, and potential therapeutic targets in the changes of higher central neural activity the cardiac cycle, and BP during the occurrence of autonomic dysfunction in α‐synucleinopathies.

HRV has long been recognized as a representative marker of autonomic nervous system function in α‐synucleinopathies.^50^ However, due to the various types of HRV parameters, the selection of specific indices is still under discussion. Frequency domain has been widely studied in PD. A meta‐analysis has shown a significant reduction in LF and HF (respectively representing sympathetic and parasympathetic nervous system functions) in individuals with PD compared to control groups.^51^ Similar frequency domain alterations have also been observed in patients with MSA.^52^ However, clinical studies have found that frequency domain indices of HRV cannot differentiate between PD patients and healthy individuals, despite significant differences in clinical autonomic scores between the two groups.^41^ This study focuses on cardiovascular regulation within autonomic function, differentiating patients based on the presence of OH, and utilizes a more comprehensive set of HRV parameters. The results indicate that another type of HRV parameters, namely time domain indices including RMSSD, pNN50, MxDMn, and SDSD, can differentiate OH patients with α‐synucleinopathy and the healthy population. This suggests that time domain holds more potential in predicting this group of patients. Specifically, RMSSD and pNN50 reflect vagal tone, MxDMn reflects the heartbeat modulation driven by the respiratory function through the vagus nerve, and SDSD represents a combined effect of sympathetic and vagal activity.^53^ Furthermore, in this study, there were strong correlations observed among nearly all HRV parameters. Both frequency domain and time domain parameters were found to be associated with the supine‐to‐standing blood pressure or heart rate differences within 5 min, both reflecting the severity of cardiovascular regulatory variability. Therefore, the occurrence of OH in α‐synucleinopathies is likely a result of combined impairments in both sympathetic and vagal nervous system functions. Additionally, the results of this study suggested that seated resting‐state HRV indicators were more sensitive than the supine‐to‐standing heart rate differences in OH patients with α‐synucleinopathies. This also indicates that the commonly used clinical practice of measuring only the changes in heart rate with body position cannot substitute for HRV. Seated resting‐state HRV, being a comfortable examination method, avoids the potential serious consequences such as imbalance, falls, syncope, or even blackout that can occur in α‐synucleinopathy patients due to rapid changes in body position.

It is well established that higher cortical activity can exert feedback and negative feedback regulation on cardiac pulsations. Previous studies have suggested that the anterior cingulate cortex^54^ and medial prefrontal cortex^55^ of visceral center trigger shorter cardiac cycles through the deinhibition of the vagus nerve. Mueller et al. proposed the cardio‐electroencephalographic covariance tracking (CECT) technique, which revealed the “N300H” phenomenon, where a larger EEG amplitude 300 ms after external stimulation corresponds to a shorter subsequent cardiac cycle.^56^ This phenomenon explains the physiological mechanism of brain‐heart coupling. Serotonin (5‐HT) plays a major role in this regulation, and consumption of its precursor weakens the N300H effect.^57^ Catecholaminergic neurons, distributed throughout the central cardiovascular regulatory system, mediate the rapid communication between the sympathetic nerves and heart.^58^ Additionally, HRV, which can reflect cardiac regulation, has also been proposed as a peripheral indicator of the integrity of the prefrontal cortical network. It can be used as a peripheral indicator to indirectly reflect higher central functions such as executive function, motor ability, and emotions.^59^ These findings support the anatomical and functional basis of research on brain‐heart interactions in this study.

Previous studies on mouse models have demonstrated a close correlation between the accumulation of synaptic proteins in neurons and the slowing of brain oscillations, as well as changes in network excitability.^60^ Under the pathological condition of α‐syn propagation, the specific cortical electrical activities related to cardiovascular regulation are not yet well‐established. The current mainstream belief is that neurogenic cardiac dysregulation in α‐synucleinopathies is a result of the deposition of α‐syn deposition in the autonomic nervous system,^61^ leading to functional disruption, from the central brainstem nuclei and spinal preganglionic neurons to peripheral postganglionic neural networks.^62^ These mechanisms contribute to the chronic and progressive impairment of cardiac regulatory capacity in α‐synucleinopathies.^63^ However, there is currently limited clinical research on the advanced cortical EEG and ECG in α‐synucleinopathy patients with abnormal blood pressure regulation. Previously, Li‐Min Liou et al.^41^ conducted a pioneering study on the central and peripheral autonomic network connectivity in patients with PD. They employed synchronous EEG and HRV spectral analysis, with clinical reference to autonomic function questionnaires. The results revealed significant correlations between the power of specific EEG frequency bands in electrode areas of F4, F7, Cz, and Pz, and the power of HRV indices in PD patients, suggesting the impact on the compensatory region associated with relative deficiencies in the fronto‐striatal circuitry. In this study, the power of beta1 waves (F4 and Fp2), beta2 waves (Fp2 and F4) and the peak frequency of theta waves (Cz) showed significant differences in the α‐OH group and α‐NOH group, indicating distinct electrical activities observed in the frontal and prefrontal regions of the non‐dominant hemisphere in the presence or absence of OH. This suggests that neurodegenerative pathological changes may have an impact on the recognized autonomic central‐executive‐insular prefrontal cortex and the surrounding brain parenchyma, further affecting its regulatory capacity in blood pressure control. This study also found that abnormal electrical activities (including theta, beta1, and beta2) in brain regions such as Fp2, F4, Cz, P3, P4, and O2 were directly or indirectly correlated with multiple cardiovascular indicators, including seated resting‐state HRV, BP, and HR change during supine and standing positions. These abnormal electrical activities, along with previous research findings, indicate that the autonomic symptoms in α‐synucleinopathies are significantly influenced by the higher cortical regions. This further suggests that the regulatory activities between the central cortex and the cardiovascular system, whether unidirectional or bidirectional, may not be limited to the previously recognized autonomic cortical centers alone. It is possible that the disease state disrupts direct influences on multiple cortical functional areas or indirect effects on complex brain network connections, leading to the loss of central compensatory mechanisms in maintaining blood pressure.

These results of this study may also provide new insights into the therapeutic targets for brain stimulation in α‐synucleinopathies, such as repetitive transcranial magnetic stimulation (rTMS) or transcranial direct current stimulation (tDCS). The medial prefrontal cortex, ventromedial prefrontal cortex, insular cortex, anterior cingulate cortex, and main subcortical nuclei were previously thought to form critical network circuits that can modulate multiple sympathetic and parasympathetic conduction pathways and affect the cardiovascular system.^64^ Additionally, Critchley et al. found that the dorsal anterior cingulate cortex, medial prefrontal cortex, insula, and medial parietal cortex are associated with high HRV through hypothalamic downregulation.^65^ This suggests that the higher cortical regions possess the functional basis for upregulating heartbeats. In a study by T. Yoshida et al., they delivered magnetic stimulation through a circular coil using repetitive rTMS on the Cz region, which increased the LF power of HRV in healthy male volunteers, indicating activation of the sympathetic nervous system.^66^ In this study, the aforementioned circuits were covered by the differential brain regions associated with cardiovascular dysregulation and the potentially correlated brain regions in α‐synucleinopathies with OH. The compensatory increase in the As value associated with alpha electrical activity in the nondominant hemisphere's occipital lobe, observed in OH group, positively correlated with the elevation in heart rate during postural changes, providing a new avenue for treatment inspiration. These findings may have implications for future studies investigating the therapeutic efficacy of targeted stimulation in autonomic dysfunction in α‐synucleinopathies.

This study is a cross‐sectional study. In subsequent research, we will continue to longitudinally track the changes in heart‐brain electrical indicators in the study population to supplement and validate the conclusions. Meanwhile, we will improve the analysis of brain imaging structure to further evaluate the changes in gross brain structure in the target population.

## 5 CONCLUSION

Lower levels of certain time domain parameters in rapid resting‐state HRV, such as MxDMn, RMSSD, SDSD, and pNN50, may predict the occurrence of OH in α‐synucleinopathy patients. Additionally, the deterioration of HRV parameters correlates with synchronous abnormal qEEG patterns, suggesting potential brain stimulation targets related to OH in α‐synucleinopathies.

## AUTHOR CONTRIBUTIONS

X.D.P. provided general overall. L.L., Y.Z.C., and P.L.H. were involved in the collection of cases and observation parameters. L.L. performed the data analysis and manuscript editing. J.J.Z. and J.H.Z. assisted in data verification. X.D.P. reviewed the manuscript. All authors provided suggestions for manuscript revisions, reviewed the final draft, and agreed to submit it for publication.

## FUNDING INFORMATION

This work was supported by grants from Joint Funds for Innovation of Science and Technology, Fujian Province (No. 2021Y9037); The Financial Special Project of Fujian Province (No. 2021XH009); The National Clinical Key Special Subject of China.

## CONFLICT OF INTEREST STATEMENT

No conflicts of interest exist.

## PATIENT CONSENT STATEMENT

All participants provided written informed consent at the time of study registration and data collection. Informed consent for participants with severe cognitive impairment was obtained from their guardians.

## PERMISSION TO REPRODUCE MATERIAL FROM OTHER SOURCES

No permission to reproduce material from other sources exist.

## CLINICAL TRIAL REGISTRATION

NCT05527067.

## Supporting information


Table S1.

## Data Availability

The data that support the findings of this study are available from the corresponding author upon reasonable request.
