# Supplementary material for: Synchronous monitoring of brain‐heart electrophysiology using heart rate variability coupled with rapid quantitative electroencephalography in orthostatic hypotension patients with α‐synucleinopathies: Rapid prediction of orthostatic hypotension and preliminary exploration of brain stimulation therapy
Source: CNS Neurosci Ther. 2024 Feb 7;30(2):e14571. doi: 10.1111/cns.14571 (PMC10850923; doi:10.1111/cns.14571)
Supplement: Supplementary file 1 — Table S1. [file CNS-30-e14571-s001.docx]

**Synchronous monitoring of brain-heart electrophysiology using heart rate variability coupled with rapid quantitative electroencephalography in orthostatic hypotension patients with α-synucleinopathies: rapid prediction of orthostatic hypotension and preliminary exploration of brain stimulation therapy**

Lin Lin^1,2,3,4, †^, Yingzhe Cheng^1,2,3,4, †^, Peilin Huang^1,2,3,4, †^, Jiejun Zhang^1,2,3,4,5^, Jiahao Zheng^1,2,3,4^, Xiaodong Pan^1,2,3,4, *^

1 Department of Neurology, Center for Cognitive Neurology, Fujian Medical University Union Hospital, NO. 29, Xinquan Road, Fuzhou City, 350001, China

2 Fujian Institute of Geriatrics, Fujian Medical University Union Hospital, NO. 29, Xinquan Road, Fuzhou City, 350001, China

3 Institute of Clinical Neurology, Fujian Medical University, NO. 29, Xinquan Road, Fuzhou City, 350001, China

4 Fujian Key Laboratory of Molecular Neurology, Fujian Medical University, NO. 88, Jiaotong Road, Fuzhou City, 350001, China

5 Center for Geriatrics, Hainan General Hospital, NO. 19, Xiuhua Road, Hainan Province, 570311, China

† Lin Lin, Yingzhe Cheng and Peilin Huang have contributed equally to this work and share first authorship.

Supplementary table 1. Demographic and medical data of patients with α-synucleinopathies included as per the eligibility criteria

PD: Parkinson's disease; MSA: multiple system atrophy; DLB: dementia with Lewy bodies. BPH: benign prostate hyperplasia; TN: thyroid nodule; CC: chronic cholecystitis.

| Patients | Age (years) | | Sex (M/F) | | Disease | Co-Morbidities | Orthostatic Hypotension (Y/N) | |
| --- | --- | --- | --- | --- | --- | --- | --- | --- |
| 1 | 70 | M | | PD | | Nasal Trauma | N |  |
| 2 | 70 | M | | PD | | n | N |  |
| 3 | 73 | M | | PD | | BPH | Y |  |
| 4 | 67 | M | | PD | | n | N |  |
| 5 | 64 | F | | PD | | n | N |  |
| 6 | 78 | F | | PD | | Sicca Syndrome | N |  |
| 7 | 64 | M | | PD | | n | Y |  |
| 8 | 68 | M | | PD | | TN | Y |  |
| 9 | 75 | F | | PD | | n | Y |  |
| 10 | 72 | F | | PD | | n | Y |  |
| 11 | 64 | F | | PD | | n | Y |  |
| 12 | 75 | M | | PD | | BPH | Y |  |
| 13 | 68 | F | | PD | | n | N |  |
| 14 | 59 | F | | PD | | n | N |  |
| 15 | 61 | M | | PD | | n | N |  |
| 16 | 77 | M | | PD | | n | Y |  |
| 17 | 77 | M | | PD | | BPH | Y |  |
| 18 | 37 | F | | PD | | n | N |  |
| 19 | 63 | M | | PD | | n | Y |  |
| 20 | 75 | F | | PD | | CC | Y |  |
| 21 | 57 | F | | PD | | n | N |  |
| 22 | 53 | F | | PD | | n | N |  |
| 23 | 63 | M | | PD | | n | N |  |
| 24 | 78 | M | | PD | | BPH | N |  |
| 25 | 60 | M | | PD | | n | Y |  |
| 26 | 69 | M | | PD | | TN | Y |  |
| 27 | 62 | M | | PD | | n | Y |  |
| 28 | 70 | M | | PD | | hernia | Y |  |
| 29 | 80 | M | | PD | | n | N |  |
| 30 | 71 | F | | PD | | n | Y |  |
| 31 | 69 | M | | PD | | n | Y |  |
| 32 | 72 | M | | PD | | n | Y |  |
| 33 | 75 | M | | PD | | BPH | Y |  |
| 34 | 74 | M | | PD | | n | Y |  |
| 35 | 47 | F | | MSA | | myoma of uterus | N |  |
| 36 | 36 | F | | MSA | | n | N |  |
| 37 | 59 | F | | MSA | | n | N |  |
| 38 | 58 | F | | MSA | | n | Y |  |
| 39 | 76 | M | | MSA | | CC | Y |  |
| 40 | 53 | F | | MSA | | n | N |  |
| 41 | 61 | F | | MSA | | cataract | Y |  |
| 42 | 61 | F | | MSA | | n | N |  |
| 43 | 47 | F | | MSA | | n | Y |  |
| 44 | 73 | F | | MSA | | n | Y |  |
| 45 | 80 | F | | MSA | | n | N |  |
| 46 | 50 | M | | MSA | | n | Y |  |
| 47 | 74 | F | | DLB | | cataract | N |  |
